# Gene expression profiling integrated into network modelling reveals heterogeneity in the mechanisms of BRCA1 tumorigenesis

**DOI:** 10.1038/sj.bjc.6605275

**Published:** 2009-10-13

**Authors:** R Fernández-Ramires, X Solé, L De Cecco, G Llort, A Cazorla, N Bonifaci, M J Garcia, T Caldés, I Blanco, M Gariboldi, M A Pierotti, M A Pujana, J Benítez, A Osorio

**Affiliations:** 1 Human Genetics Group Human Cancer Genetics Program, Spanish National Cancer Center (CNIO) and CIBERER, Melchor Fernández Almagro, 3, Madrid 28029, Spain; 2 Biostatistics and Bioinformatics Unit, and Translational Research Laboratory, Catalan Institute of Oncology, IDIBELL, L'Hospitalet, Av. Gran Via s/n km, 2,7, Barcelona 08907, Spain; 3 Fondazione Istituto Nazionale dei Tumori, Milan, Italy; 4 Fondazione Istituto FIRC Oncologia Molecolare, Via Giacomo Venezian 1, Milan, Milano 20133, Italy; 5 Genetic Counseling Unit, Catalan Institute of Oncology, IDIBELL, L'Hospitalet, Av. Gran Via s/n km, 2,7, Barcelona 08907, Spain; 6 Department of Pathology, Fundación Jiménez Díaz, Avda. Reyes Católicos, 2–28040 Madrid, Spain; 7 Clinical Oncology Laboratory, Hospital Clínico San Carlos, Profesor Martin Lagos s/n E-28040, Madrid, Spain

**Keywords:** gene expression profiling, BRCA1-associated tumours, prognosis

## Abstract

**Background:**

Gene expression profiling has distinguished sporadic breast tumour classes with genetic and clinical differences. Less is known about the molecular classification of familial breast tumours, which are generally considered to be less heterogeneous. Here, we describe molecular signatures that define BRCA1 subclasses depending on the expression of the gene encoding for oestrogen receptor, *ESR1*.

**Methods:**

For this purpose, we have used the Oncochip v2, a cancer-related cDNA microarray to analyze 14 BRCA1-associated breast tumours.

**Results:**

Signatures were found to be molecularly associated with different biological processes and transcriptional regulatory programs. The signature of *ESR1*-positive tumours was mainly linked to cell proliferation and regulated by ER, whereas the signature of *ESR1*-negative tumours was mainly linked to the immune response and possibly regulated by transcription factors of the REL/NF*κ*B family. These signatures were then verified in an independent series of familial and sporadic breast tumours, which revealed a possible prognostic value for each subclass. Over-expression of immune response genes seems to be a common feature of ER-negative sporadic and familial breast cancer and may be associated with good prognosis. Interestingly, the *ESR1*-negative tumours were substratified into two groups presenting slight differences in the magnitude of the expression of immune response transcripts and REL/NF*κ*B transcription factors, which could be dependent on the type of *BRCA1* germline mutation.

**Conclusion:**

This study reveals the molecular complexity of BRCA1 breast tumours, which are found to display similarities to sporadic tumours, and suggests possible prognostic implications.

Breast cancer is a complex disease, encompassed by different clinically and molecularly stratified entities. In 2000, Perou and colleagues demonstrated that tumour phenotypic diversity correlates with differences in global gene expression patterns, which in turn reflect aspects of the biological behaviour of the tumours (Perou *et al*, 2000). This study and subsequent ones (Sorlie *et al*, 2001; van‘t Veer *et al*, 2002; Bertucci *et al*, 2006) provide detailed analysis of correlations with histopathological and clinical characteristics.

The level of expression of the oestrogen receptor (ER) is a key feature that divides breast tumours into two main clusters. ER-positive tumours include the luminal A and luminal B subclasses showing different prognosis (Perou *et al*, 2000). Tumours with very low or no detectable expression of ER can be classified into HER2/ErbB2-positive, normal breast-like and basal-like (Perou *et al*, 2000; Sorlie *et al*, 2001). The first subclass is characterised by over-expression of *ERBB2* and other genes at the 17q22 amplicon. Normal breast-like tumours show high heterogeneity, with expression of genes related to the adipose tissue and other nonepithelial cells (Sorlie *et al*, 2001). Finally, the basal-like subclass is known to be negative for HER2/ErbB2, ER and the progesterone receptor (PR), and characterised by the expression of genes from the basal epithelium with high frequency of *TP53* mutations (Sorlie *et al*, 2001; Foulkes *et al*, 2004; Bertucci *et al*, 2006; Turner and Reis-Filho, 2006; Yehiely *et al*, 2006; Adelaide *et al*, 2007; Jumppanen *et al*, 2007). Basal-like tumours account for up to 15% of all breast cancers and the clinical handling of this subclass is a major challenge, once they do not respond to conventional targeted therapies.

Similar features in familial breast cancer are less clearly understood, partially due to the fact that very few studies have been published regarding expression profiling of the corresponding breast tumours. This lack of information probably lies in the difficulty to collect frozen tumours from hereditary breast cancer cases genetically characterised. This is also reflected in the small size of the series that have been published so far. In 2001, Hedenfalk *et al* (2001) examined a small series of tumours from patients with germline mutations in *BRCA1* or *BRCA2* genes and differentiated two groups within BRCA1. They suggested that the DNA repair and apoptosis pathways were altered in the BRCA1 tumours and that, although most of these were ER negative, ER status alone was not sufficient to discriminate both classes. In a subsequent study, Hedenfalk *et al* (2003) proposed novel classes for the familial non-BRCA1/BRCA2 breast tumours and a different expression profile to those of the BRCA1- and BRCA2-associated tumours reported earlier (Hedenfalk *et al*, 2003).

More recently, it has been suggested that most tumours arising in *BRCA1* mutation carriers display a basal-like phenotype, with the percentages reported ranging from 44 to almost 100% (Diaz *et al*, 2007; Melchor and Benitez, 2008). It is not yet known whether there are differences in the molecular or clinical characteristics within BRCA1 or between BRCA1 and sporadic basal-like tumours. Two recent expression profiling studies have revealed further stratification of the sporadic ER-negative breast tumours (Kreike *et al*, 2007; Teschendorff *et al*, 2007). Kreike *et al* (2007) reported that basal-like tumours can be divided into five different subclasses and linked the presence of lymphocytic infiltrate and central fibrotic zones to lower risk of metastasis. In addition, Teschendorff *et al* (2007) defined four ER-negative subgroups whose clinical outcomes differ according to the expression of genes of the immune response.

Here, we used expression profiling to classify the BRCA1 breast tumours and applied an integrative approach to examine biological dependencies and differences. Tumours were initially segregated according to the expression of *ESR1* gene or the expression of basal markers. Detailed examination of the profiles of apparently uniform classes revealed molecular differences within both the *ESR1*-positive and *ESR1*-negative tumours. These subclasses were corroborated in an independent series of familial and sporadic breast tumours, which revealed possible prognostic value. We suggest that BRCA1 breast tumours show a high degree of molecular complexity and define the wiring diagram of signalling pathways involved in their tumorigenesis.

## Materials and methods

### Tumoral tissues

Fourteen frozen tumours from patients harbouring germline mutations in *BRCA1* were used (Supplementary Table S4). Samples proceeded from CNIO (Madrid), Istituto Tumori (Milan), Hospital Clínico San Carlos (Madrid) and ICO (Barcelona). Patient selection and mutational analysis are described elsewhere (Diez *et al*, 2003; Manoukian *et al*, 2007). A tissue microarray containing an independent series of 15 BRCA1 tumours previously classified as basal or luminal-like phenotype (Palacios *et al*, 2005) was used to analyse the CD133 (Prominin 1) and MMP7 (Matrix Metalloproteinase 7) proteins. Immunohistochemical staining was performed by the Labelled Streptavidin-Biotin method (Dako, Glostrup, Denmark) with a heat-induced antigen retrieval step. Immunofluorescence was performed with a fluorescence-labelled secondary antibody (Alexa 488 for MMP7 and Alexa 555 for CD133).

### Evaluation of tumour samples

Frozen tumour samples were included in a polyvinyl matrix (OCT). A slice of each sample was stained with hematoxilin-eosin and examined by a pathologist to determine the amount of tumoral cells; those with a tumour content >70% were used for subsequent RNA extraction.

### RNA extraction, cDNA labelling and hybridisation

Total RNA extraction was performed (TRIZOL, Invitrogen, Carlsbad, CA, USA) and mRNA was amplified from 5 *μ*g of total RNA (SuperScript II, Invitrogen and Ambion, Austin, TX, USA). A new cDNA was synthesised from the amplified mRNA and labelled with Cy5. The same process was carried out with the Universal Human Reference RNA (Stratagene, La Jolla, CA, USA), which was labelled with Cy3. Hybridisation was performed onto the CNIO human OncoChip V2 following standard conditions (Tracey *et al*, 2002).

### Quantitative RT–PCR

DNA-free total RNA was obtained from a set of 10 breast cancer cell lines including the BRCA1-mutant cell line MDA-MB-436 (previously hybridised onto the Oncochip V2). One microgram of RNA was reverse transcribed using MMLV retrotranscriptase (Invitrogen) and random hexamers. Quantitative PCR assays were set up in triplicate for the *BRCA1* target gene and two control genes (*BACT* and *MRPL19*). Assays were designed using the Roche Applied Science Universal Probe Library web site for *BRCA1* (probe 11) and *MRPL19* (probe 42). *BACT* primers and probe are described elsewhere. The relative expression of *BRCA1* was determined using the free access qBase software (Hellemans *et al*, 2007), which is based on a modification of the classic delta–delta Ct method that allows for PCR efficiency correction and multiple control gene normalisation.

### Microarray data analyses

Two channel ratios (Cy5/Cy3) for each spot were generated and quantified using GenePix Pro 5.1 (Axon Instruments, Inc., Union City, CA, USA). Data were normalised with the print-tip loess method (Yang *et al*, 2002) and log2-transformed values, and filtered using the PREPROCESSOR tool (Montaner *et al*, 2006). Differentially expressed genes were declared after applying a two-tail *t*-test with the *P*-values adjusted for the false discovery rate (FDR) using the Tibshirani and Efron approach (Tibshirani and Efron, 2002). Representation of Gene Ontology (GO) terms was examined using the Onto-Express tool (Khatri *et al*, 2007), with the reference including all genes in the array that passed quality filters and with *P*-values calculated based on the hypergeometric distribution and corrected using the FDR approach. Unsupervised clustering was performed in the R programming language (82) using the Euclidean distance and Ward's minimum variance method, except in the analysis of basal markers, which was performed with the correlation and average-linkage clustering method. A bootstrapping resampling approach in the R library pvclust (Suzuki and Shimodaira, 2006) was applied to assess clustering robustness. Data from van‘t Veer *et al* (2002) were log2-transformed and cross-mapped with our gene lists using Entrez gene identifiers manually curated for all possible probes matching each gene. Histopathological and clinical information was downloaded from the publication site.

### Transcription factor and interactome analyses

Examination of predicted transcription factor binding sites was performed using the oPOSSUM tool (Ho Sui *et al*, 2005), with promoters defined as −2 kilobases (kb) to the start of transcription, and using JASPAR annotations with a matrix match threshold of 80%. Fisher's exact test was used to examine the results for over-representation and using the *Bonferroni* correction taking into account the number of motifs analysed (*n*=111). Experimentally identified binding sites for ER and E2F1 were taken from the original publications or relevant databases (Carroll *et al*, 2006; Jin *et al*, 2006). We assigned ER *cis*-regulators to the closest known gene locus (5′ end) in the May 2004 version of the human genome in the UCSC Genome Browser. The human interactome network was built by combining three previously published data sets, which consist mainly of experimentally verified interactions. The data set based on the Human Protein Reference Database (Gandhi *et al*, 2006) contains compiled and filtered binary protein interactions from most currently available databases. High-confidence yeast two-hybrid interactions from Pujana *et al* (2007) and Stelzl *et al* (2005) were then incorporated, and orthology-based predictions and homodimers were excluded to avoid bias. Proteins with no assigned Entrez GeneID were also excluded from the analyses. Shortest distances were calculated using only the giant network component and the geodesic formulation given by Freeman (Stelzl *et al*, 2005) using the R programming language (82). Differences between distributions of shortest paths were assessed with the Mann–Whitney *U-*test and GO term representations evaluated with the Onto-Express tool (Khatri *et al*, 2007), taking as a reference the protein set of the giant network component.

## Results

### *ESR1*-status or the basal-like phenotype as major classifiers

**Figure 1:**
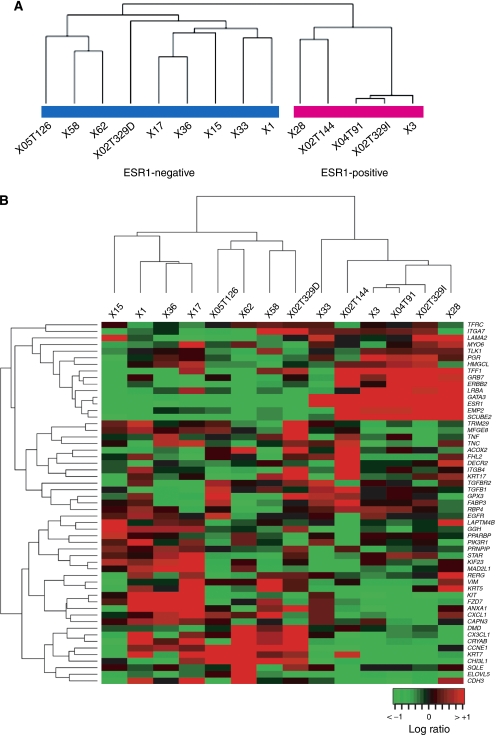
(**A**) Dendrogram resulting of unsupervised hierarchical clustering of the 14 BRCA1 tumours and 5570 genes that remained after data quality filtering. Two main clusters are observed mediated by ESR1 expression (left branch encompasses tumours over-expressing ESR1 and right branch those with modest or no detectable levels of ESR1). (**B**) Unsupervised hierarchical clustering of the 14 BRCA1 tumours using the transcript levels of 55 genes representing markers of the five distinct subclasses of sporadic breast tumours (Perou *et al*, 2000). The same clusters as in the previous analysis are observed except for sample #33.

Unsupervised clustering of the 5570 genes that remained after data filtering (see Materials and Methods) stratified the whole series of 14 BRCA1 tumours into two main branches (Figure 1A). This clustering was clearly mediated by *ESR1* expression: one cluster (five samples, right branch) over-expressed this gene whereas the rest (nine samples, left branch) mostly showed very modest or undetectable *ESR1* expression.

Examination of the ‘intrinsic gene list’ (Perou *et al*, 2000; Hu *et al*, 2006), and an exhaustive review of the literature (Perou *et al*, 2000; Sorlie *et al*, 2003) identified a set of 55 established markers present in our platform that were subsequently used for classification (Figure 1B). Two main groups were identified, one over-expressing markers typically found in sporadic luminal tumours such as *GATA3*, *TFF1* and *SCUBE2* (right panel), and the other negative for *ESR1*, *ERBB2* and the PR gene (*PGR*) (triple negative) and over-expressing genes from the basal layer such as *CDH3*, *CRYAB* and *KRT5-17* (left panel). This clustering showed consistent results with the previous classification with the exception of sample #33 that shared characteristics of both basal and luminal classes. As the classification with the 55 markers was in agreement with the immunohistochemical data for this sample (ER and PGR positive, Supplementary Table S4) we decided to maintain it within the *ESR1*-positive class.

### BRCA1 tumour subclasses and signatures

Using the set of 5570 genes that passed filtering criteria (see Materials and Methods for data quality evaluation), we examined differential expression within the BRCA1 classes relative to the common reference. Applying a FDR of 1 out of 1000, 212 genes were differentially expressed within the class of *ESR1*-positive tumours (hereafter, gene-set #1) and 670 genes differentially expressed within the class of *ESR1*-negative tumours (gene-set #2) (Supplementary Tables S1 and S2, respectively).

Biological differences between the two gene-sets were revealed by analysing GO term annotations (see Materials and Methods). Taking into account the number of genes annotated for each term and the 5570 genes, the top-ranked biological processes in gene-set #1 were transcription, DNA-dependent and cell cycle, whereas the top-ranked in gene-set #2 were the immune response and cell cycle. Consistent with the association with cell proliferation (Butt *et al*, 2007), response to oestrogen stimulus was found to be over-represented in gene-set #1 but not in gene-set #2. In addition, analysis of functional genomic data of the ER-positive cell line MCF7 (Carroll *et al*, 2006) identified a higher than expected number of genes in gene-set #1 to be transcriptionally regulated by the ER signalling pathway. Thirty-three (15%) genes in this set showed significant transcriptional changes on ER signalling and chromatin immunoprecipitation assays identified a *cis*-ER-binding site in a further 60 (28%). Finally, the gene-set #2 also contained several tumour suppressors (*EPHA3* and *EPHB2*) and proto-oncogenes (*AKT1*, *AURKA*, *ETV6*, *MITF* and *PIK3CA*), which expands on the observed enrichment of the immune response and suggests that this gene-set has a critical role in breast tumorigenesis. Together, this analysis highlights candidate biological processes that are critical in BRCA1 tumorigenesis beyond the potential of cell proliferation.

### Markers that differentiate *ESR1*-negative and *ESR1*-positive BRCA1 tumours

**Figure 2:**
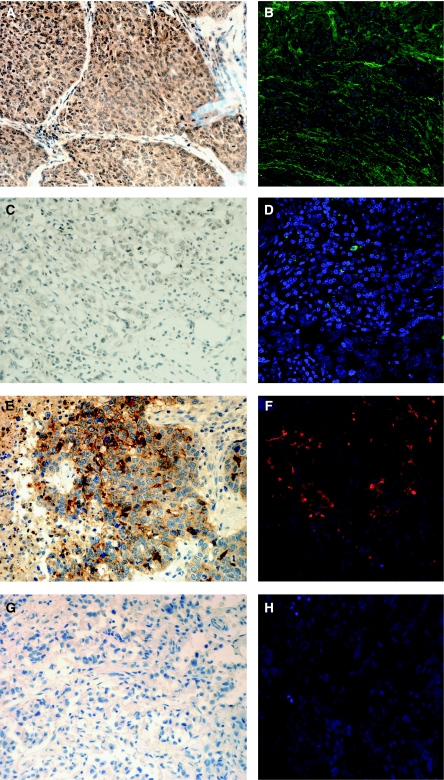
Validation through immunohistochemical (IHC) and immunofluorescense (IF) assays of the expression results obtained for the MMP7 and CD133 genes. (**A** and **B**) BRCA1 ER-negative tumour over-expressing MMP7. (**C** and **D**) BRCA1 ER-positive tumour lacking MMP7 expression. (**E** and **F**) BRCA1 ER-negative tumour over-expressing CD133. (**G** and **H**) BRCA1 ER-positive tumour lacking CD133 expression.

To identify markers that more strongly differentiate *ESR1*-based BRCA1 classes, we compared gene-sets #1 and #2 using a fold-change threshold ⩾2 in absolute value (FDR <5%). A total of 31 genes met these criteria (Table 1) and, among these, two of the most differentially expressed, *CD133* and *MMP7*, were selected for evaluation in an independent series of BRCA1 tumours (see Materials and Methods). Immunohistochemical and immunofluorescence assays of both proteins strongly correlated with the gene expression results (Figure 2): MMP7 showed strong staining in ∼80% (9 out of 11) of the ER-negative tumours but in none of the ER-positive tumours (0 out of 4), and CD133 showed strong staining in 90% of the ER-negative tumours (10 out of 11) but was completely absent from the four ER-positive tumours (*P*-values=0.011 and 0.004, respectively).

### Putative prognostic value of the immune response

**Figure 3:**
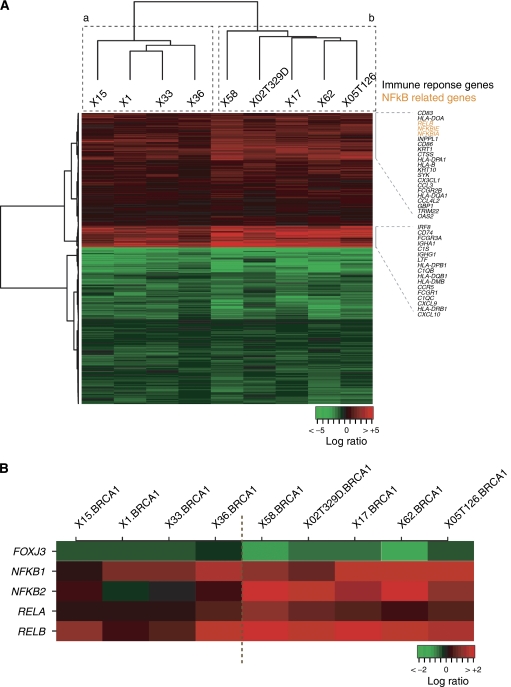
(**A**) Unsupervised hierarchical clustering of the nine ESR1-negative BRCA1 tumours using the gene-set #2. Two subgroup/class/clusters, **a** (left branch) and **b** (right branch), are observed, showing differences in the magnitude of expression of genes related to the immune response. (**B**) Unsupervised hierarchical clustering of the nine *ESR1*-negative BRCA1 tumours using transcription factor genes of the REL/NF*κ*B family. The same subgroup/class/clusters (**a** and **b**) as those found using gene-set #2 are observed.

The association between gene-set #2 and the immune response was narrowed down using child GO terms such as Cytokine and Chemokine Mediated Signalling Pathway or Lymphocyte Proliferation. Overall, 52 genes in gene-set #2 with annotations from these processes were identified and most were over-expressed in the *ESR1*-negative BRCA1 tumours. Next, using gene-set #2, an unsupervised clustering of these tumours identified two possible subclasses distinguished by the expression level of several transcripts in these processes (Figure 3A).

The expression level of immune response genes has recently been shown to provide prognostic value for sporadic ER-negative breast tumours (Teschendorff *et al*, 2007) and this biological process seems to be commonly present in breast cancer prognosis signatures (Reyal *et al*, 2008). Examination of gene-set #2 and the signature of Teschendorff *et al* (2007) identified 18 genes in common, which is higher than the number expected by chance (*P*-value <10^−7^). In agreement with this observation, differential expression of several genes in set #2 has also been associated with breast cancer metastasis, to the bone (*CX3CL1*, *FARSLA*, *FST*, *GBP2*, *HLADPA1*, *HLADPB1*, *HLADRB1*, *MITF*, *NEDD4L*, *SERPINA1* and *TGFBI*) (Kang *et al*, 2005) or to the lung (*ALDH3A1*, *COL1A1*, *EFEMP1*, *GSN*, *HLADPA1*, *HLADPB1*, *MAN1A1*, *PTPRN2* and *TNC*) (Minn *et al*, 2005). In both metastatic conditions, the number of genes in common with gene-set #2 is higher than randomly expected (*P*-values <0.05). This suggests the putative association between gene-set #2 and BRCA1 prognosis. Although the immune response gene-set is differentially expressed in the same direction in all *ESR1*-negative BRCA1 tumours examined here, it can stratify tumours in at least two additional subclasses (Figure 3a and b) depending on the magnitude of the expression of specific genes (Supplementary Table S5). The transcript levels of genes that overlap with the good prognostic signature of sporadic ER-negative tumours were higher in class B.

### Examination of independent series of ER-negative BRCA1 tumours

**Figure 4:**
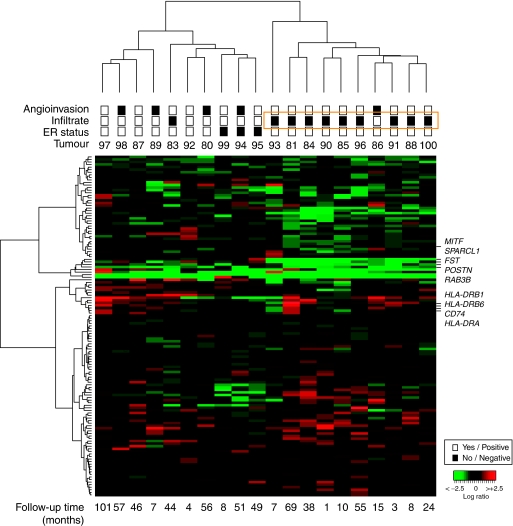
Unsupervised hierarchical clustering obtained with 94 genes differentially expressed in the ESR1-negative tumours, using the *Bonferroni* correction, in 18 BRCA1 and two BRCA2 (#99 and #94) tumours from the external series of van ‘t Veer *et al* (2002). Clustering of tumours according to the presence or not of lymphocytic infiltrate and angioinvasion is observed. Relevant genes of breast cancer metastasis and those that more strongly differentiate clusters are shown. Pathological characteristics are annotated as shown in the inset and follow-up times (months) are shown at the bottom. Expression values are represented as log2 ratios.

The observed association with the immune response and with metastasis genes indicated a possible prognostic value of the expression profiles of gene-set #2. We then examined the profiles and their association with histopathological or clinical variables in an independent series of ER-negative BRCA1 tumours (van‘t Veer *et al*, 2002). Unsupervised clustering using genes differentially expressed in our series of ER-negative tumours with *Bonferroni* correction (*n*=94) (Supplementary Table S3) classified tumours highly according to the presence of lymphocytic infiltrate, as described by van‘t Veer *et al*, 2002. This observation is consistent with the differential expression of immune response genes and highlighted those genes with greater expression differences in this condition (Figure 4). Tumour subclasses differed considerably in terms of presence/absence of angioinvasion, which leads us to speculate that these genes have a function in the prognosis of ER-negative BRCA1 breast cancer. Overall, the results for the independent series support the putative biological significance of the immune response in the molecular and clinical classification of BRCA1 tumours.

### Examination of independent series of ER-positive tumours

The expression levels of transcripts represented in gene-set #1 (*ESR1*-positive BRCA1 tumours) were examined in an independent series of 56 sporadic ER-positive breast tumours (van‘t Veer *et al*, 2002). This analysis identified a group of tumours with good prognosis, almost all of which were of low grade and metastasis-free up to 5 years (Supplementary Figure S1). These good prognosis tumours were mainly characterised by over-expression of *ERBB3* and down-regulation of cell cycle-related genes such as *CCNA2* and *CCNB2*. Notably, *ERBB3* expression has recently been associated with favorable clinical outcome of invasive ductal carcinomas (Lee *et al*, 2007).

### Transcriptional regulation of BRCA1 signatures and association with the type of germline mutation

To investigate the higher-order regulation of BRCA1 signatures we combined the analysis of predicted transcription factor binding sites with the examination of differential expression and profiles in our series. From the analyses of ER functional genomic data shown above, it was demonstrated that many genes in set #1 were regulated directly or indirectly by ER. Examination of JASPAR transcription factor motifs in the promoters of genes in set #2 indicated over-representation of predicted binding sites of C/EBP and RELA (*Bonferroni* corrected Fisher's exact test *P*-values <0.05). Two transcription factors of this family present in our array, *NFκB2* and *RELB*, showed over-expression in *ESR1*-negative BRCA1 tumours with fold changes relative to *ESR1*-positive tumours ranging from 3.7 to 4.1, respectively (two-tailed *t*-test *P*-values <0.05.

Consistent with these observations, the expression levels of *REL*/*NFκB* transcription factors classified our *ESR1*-negative BRCA1 tumours in the same way as observed for the complete gene-set #2, with two main subclasses (a and b) that differed in the magnitude of expression change of immune response genes (but not in the direction) (Figure 3B). Importantly, BRCA1 has been identified as interacting directly with RELA and, thus, activating NF*κ*B target genes (Benezra *et al*, 2003). We then examined the association between expression profiles and BRCA1 mutation types in our series. All of the *ESR1*-negative BRCA1 tumours with low expression of *NFκB* originated from BRCA1 truncating mutations that probably led to a complete absence of the protein through the nonsense-mediated mRNA decay mechanism (NMD) (Supplementary Table S4) (Perrin-Vidoz *et al*, 2002). In contrast, three of the five BRCA1 *ESR1*-negative tumours, which showed the highest levels of *NFκB2* and *RELB* expression, harboured missense mutations that might led to an aberrant but still present BRCA1 protein.

To evaluate these observations, we examined the expression levels of 72 NF*κ*B target genes related to apoptosis and the immune system (http://people.bu.edu/gilmore/NFkB/target/index.html) in our ER-negative BRCA1 tumours and in the BRCA1-mutated cell line MDA-MB-436, showing very low levels of the BRCA1 transcript with respect to the BRCA1-mutated cell line HCC1937 (harbouring a mutation that is known not to trigger NMD) and other control breast cancer cell lines. Two clusters were again observed according to the expression of target genes, as described above, and MDA-MB-436 was classified within the cluster of tumours showing low expression of NF*κ*B targets (Supplementary Figure S2).

### Higher-level study of BRCA1 signatures

**Figure 5:**
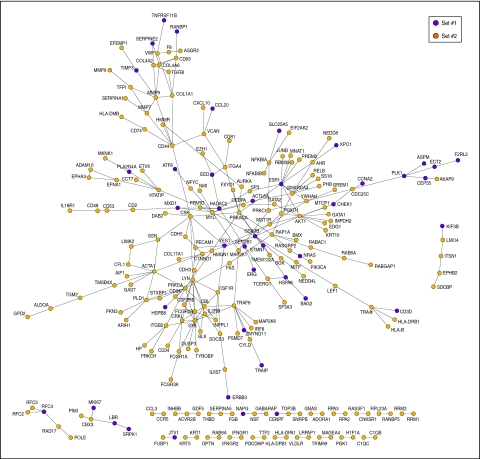
Interactome network of proteins encoded by the gene-sets #1 and #2 as shown in the inset.

To better understand BRCA1 signatures and their role in breast tumorigenesis, we examined them in the context of the network of human protein–protein interactions or interactome network. This analysis revealed that many proteins encoded by these signatures are included in a giant component with 235 nodes or proteins and 532 edges or interactions (Figure 5), which suggests that the gene products of gene-sets #1 and #2 work in functionally related pathways or processes.

Functional relationships between proteins can be defined as direct interactions, complex memberships or relatively close connections in the interactome network. To determine the exact relationships between the proteins considered here, interactome paths were calculated between proteins encoded by gene-sets #1 and #2 and compared with the complete distribution of shortest paths in the giant component. The shortest paths identified between signatures were smaller than those in the giant component (Mann–Whitney *U*-test *P*-value <0.001), which supports the proposed existence of functional and dynamical relationships between BRCA1 signatures.

To further examine the functional association between the signatures, we analysed the representation of GO biological process terms in their interactome network neighbourhoods (i.e. proteins that interact directly with each signature, but excluding proteins that belong to any signature). This analysis confirmed previously observed over-representation of certain processes in differentially expressed genes: gene-set #1 showed significant over-representation of neighbours involved in the Steroid Hormone Receptor Signalling Pathway, the Cell Cycle and Cell Death, but not in the Immune Response; and gene-set #2 showed significant over-representation of all these processes except the Steroid Hormone Receptor Signalling Pathway (Table 2). These observations show the impact of gene expression changes on protein associations mediating BRCA1 tumorigenesis.

## Discussion

In this study, we analysed the expression profiling of a series of 14 BRCA1 tumours, which gave insights into the biological processes and molecular wiring diagrams that mediate tumorigenesis. Although the sample size is small, it is worth to note the difficulty of collecting frozen tumour samples from familial breast cancer cases genetically characterised. This is probably the cause of the very few studies published so far regarding the expression profiling of these tumours, all of them using sample sizes similar to ours (Hedenfalk *et al*, 2001; van‘t Veer *et al*, 2002). Sixty percent of the tumours analysed here showed low or no expression of *ESR1* or over-expressed genes typically found in basal-like tumours, whereas the remaining tumours showed over-expression of *ESR1* and a nonbasal-like phenotype. The results are in agreement with other series in which the percentage of BRCA1 breast tumours showing a basal phenotype defined by immunohistochemical markers ranges from 44% to almost 100% (Diaz *et al*, 2007).

### Signatures associated with BRCA1 tumours

In addition to the major classification mediated by *ESR1* or basal markers, this study reveals further complexity of BRCA1 tumours. Over-representation of transcription DNA-dependent and cell cycle-related genes and transcripts directly or indirectly regulated by ER was detected in genes differentially expressed in *ESR1*-positive BRCA1 tumours. As cell proliferation-related processes and the ER signalling pathway are known to have an important function in breast cancer prognosis (Yager and Davidson, 2006), we analysed the expression levels of gene-set #1 in an external series of 56 ER-positive sporadic breast tumours (van‘t Veer *et al*, 2002). Unsupervised analysis identified a subclass of tumours of which the majority did not show angioinvasion or metastases (Supplementary Figure S1). This subclass was characterised by under-expression of cell-cycle or proliferation-associated genes such as *CCNA2* and *CCNB2*. As stated above, these features could explain the less aggressive behaviour of these tumours. An interesting gene over-expressed in gene-set #1 was *ERBB3*. Recently, Lee *et al* (2007) used immunohistochemistry to analyse 378 sporadic invasive ductal carcinomas. They found an association between *ERBB3* expression and positive hormonal receptors status, and an inverse correlation with histological grade, which is consistent with our findings. Thus, *ERBB3* could be not only a predictor of good prognosis within ER-positive sporadic and familial breast cancer patients but also a putative therapeutic target for these tumours.

Analysis of genes differentially expressed in *ESR1-*negative BRCA1 tumours revealed over-representation of genes involved in the immune response and cell cycle. Teschendorff *et al* (2007) described five tumour subclasses within the ER-negative class that can be distinguished according to the patterns of four gene expression clusters associated with cell cycle, immune response, extracellular matrix and steroid hormone response. These clusters were related to clinical outcome, and an association was observed between good prognosis and over-expression of immune response genes, independently of the presence of lymphocytic infiltrate. In the same study, the authors analysed 18 BRCA1 tumours from van‘t Veer *et al* (2002) and found that they were most similar to the cell cycle-positive and immune response-positive subclass. In agreement with these findings, the two most commonly represented pathways in our *ESR1*-negative BRCA1 subclass were the immune response and the cell cycle. In addition, analysis of gene-set #2 in *ESR1*-negative tumours revealed two probable subclasses distinguished by differences in the magnitude of the expression change of immune response genes that may link to differences in prognosis. Additional research, however, in larger and independent series is needed to further elucidate this relationship.

Using a similar approach as for gene-set #1, we analysed gene-set #2 in the 18 BRCA1 tumours of van‘t Veer *et al* (2002), finding that the signature was able to classify the samples according to the presence of infiltrate and absence of angioinvasion (Figure 4). Lymphocytic infiltrate is known to be associated with good prognosis (Lee *et al*, 2006), and a recent study by Kreike *et al* (2007) focused on triple-negative sporadic breast tumours revealed that 5-year metastasis-free survival in patients with a moderate or large amount of lymphocytic infiltrate in their tumours was 88%, which was higher than for those with minimal or no lymphocytic infiltrate. In our case it was not possible to determine whether the signature could predict patient outcome independently of infiltrate status, due to the almost perfect correlation between the presence of infiltrate and absence of angioinvasion, but the same results in an independent series support the biological significance of the immune response in the classification of ER-negative BRCA1 tumours.

Two of the 31 most differentially expressed genes between the ESR1-negative and ESR1-positive BRCA1 tumours, *CD133* and *MMP7* (Table 1), were evaluated by immunohistochemistry and immunofluorescence in an independent series of 15 BRCA1 tumours, previously classified as basal or luminal (Figure 2). There was good agreement between mRNA and protein levels for both genes (*P*-value=0.011 and 0.004 for MMP7 and CD133, respectively), supporting our results.

### Involvement of NF*κ*B in the regulation of BRCA1 signatures

We combined the prediction of transcription factor binding sites and the examination of differential expression to determine which transcription factors could be driving the signatures. The ER-negative BRCA1 profiles may be regulated by the NF*κ*B complex. Over-expression of two genes from this family, *NFκB2* and *RELB*, was then observed consistent with motif predictions (Figure 3B). Nuclear Factor-kB is a ubiquitous transcription factor that coordinates several gene products such as cell adhesion molecules, chemokines, cytokines, growth factors and regulators of apoptosis (Chen *et al*, 1999). Over-expression of NF*κ*B subunits NF*κ*B3 and NF*κ*B1 were found in a high percentage of breast tumours and are inversely correlated with ER-status (Wang *et al*, 2007), which is in agreement with our findings. Activation of the NF*κ*B pathway has been mainly related to tumour promotion; however, negative effect on tumour development has also been reported, especially in epithelial cells that has lead to the idea that NF*κ*B can either promote or oppose tumour development (Karin, 2006).

In our series the over-expression of genes related to immune response is one of the intrinsic characteristics of ESR1-negative BRCA1 tumours. The expression levels of these genes can be used to stratify them into two different subgroup/class/clusters (A and B), which were classified according to the expression levels of REL/NF*κ*B genes, and a high correlation was also observed with the expression of apoptosis genes regulated by them such as PYCARD, BCL2A1, CASP4, TRAF1 and TRAF2 and other NF*κ*B target genes such as those involved in transcription and the immunological cascade (CCR5, CD48, NF*κ*B2 and RELB).

It has been suggested that BRCA1 acts as a co-activator of NF*κ*B (Benezra *et al*, 2003). It is worth noting that all of the *ESR1*-negative BRCA1 tumours in group A, which show low expression of *NFκB*, harboured truncating mutations in the central portion of *BRCA1* that are thought to trigger the nonsense-mediated mRNA decay mechanism (Perrin-Vidoz *et al*, 2002) (Supplementary Table S4). Interestingly, the BRCA1 cell line MDA-MB-436, which showed lower levels of the *BRCA1* transcript than those found in control breast cancer cell lines measured by quantitative RT–PCR (data not shown), had the same pattern of NF*κ*B-related gene expression as the *ESR1*-negative-A tumours. In contrast, 60% of the *ESR1*-negative tumours (group B), which show the highest expression of *NFκB2* and *RELB*, harboured missense mutations in *BRCA1*, which could produce aberrant proteins but conserving some activities such as the capacity to activate the NF*κ*B machinery. Given the limited size of our series, whether these findings are stable and could be relevant to the prognosis of *BRCA1* mutation carriers depending on the activation of NF*κ*B target genes should be further analysed in a larger sample set.

## Conclusions

In summary, in this study we have established the gene expression profiling of a series of BRCA1 tumours and found that there is a further degree of heterogeneity beyond the main classification by the expression of *ESR1* and the presence or absence of a basal-like phenotype. We have identified specific signatures for *ESR1-*positive and *ESR1*-negative BRCA1 tumours, the latter characterised by the enrichment of immune response and cell-cycle genes, and have found that slight differences in the level of expression of the immune response stratify the ER-negative BRCA1 tumours into two additional subgroups (A and B). NF*κ*B could be a major driver responsible for the levels of both immune response and apoptotic genes in this tumour group/class/cluster.

## Figures and Tables

**Table 1:** Description of the 31 genes that more strongly differentiate *ESR1*-based BRCA1 subclasses using a fold-change threshold higher or lower than 2 or −2, respectively, and a FDR lower than 5%

**Gene name (HUGO)**	**Description**	**Chromosome**	**Start (bp)**	**End (bp)**	**Strand**	**Fold change**	**FDR *P*-value**
*C10orf116*	Chromosome 10 open reading frame 116	10	88718168	88720646	+	3.77	6.4207E-04
*CD133*	Prominin 1	4	15578950	15694453	−	−2.70	1.5661E-02
*CRABP2*	Cellular retinoic acid binding protein 2	1	154936034	154942232	−	2.12	7.7835E-03
*ERBB3*	V-erb-b2 erythroblastic leukemia viral oncogene homolog 3 (avian)	12	54760159	54783395	+	2.13	1.0152E-05
*ESR1*	Estrogen receptor 1	6	152168512	152466099	+	4.25	4.4951E-04
*F2RL2*	Coagulation factor II (thrombin) receptor-like 2	5	75947064	75954996	−	2.12	8.3191E-03
*GATA3*	GATA binding protein 3	10	8136673	8157170	+	3.30	1.3560E-03
*GPR98*	G protein-coupled receptor 98	5	89945799	90495789	+	3.40	6.0975E-03
*HIST1H1C*	Histone cluster 1, H1c	6	26163894	26164678	−	2.49	1.6326E-03
*HSPB8*	Heat shock 22 kDa protein 8	12	118100978	118116933	+	2.86	4.4951E-04
*LAMP3*	Lysosomal-associated membrane protein 3	3	184322698	184363317	−	−2.29	9.6204E-03
*LDHB*	Lactate dehydrogenase B	12	21679543	21702043	−	−2.32	6.4207E-04
*LMO4*	LIM domain only 4	1	87566739	87587021	+	−2.42	1.3021E-03
*LOC124220*	Similar to common salivary protein 1	16	2820174	2822285	+	3.78	4.8845E-04
*LOC391271*	Hypothetical LOC391271	21	20719132	20720350	−	2.65	9.3351E-05
*MAML2*	Mastermind-like 2 (Drosophila)	11	95349407	95355511	−	−2.14	1.0648E-02
*MID1*	Midline 1 (Opitz/BBB syndrome)	1	145841560	145848017	+	−2.07	2.1773E-02
*MMP7*	Matrix metallopeptidase 7 (matrilysin, uterine)	11	101896450	101906688	−	−2.85	7.1351E-03
*PRAME*	Preferentially expressed antigen in melanoma	22	21220124	21231696	−	−4.30	4.3286E-04
*PSAT1*	Phosphoserine aminotransferase 1	9	80101879	80134827	+	−2.11	3.2517E-03
*REEP5*	Receptor accessory protein 5	5	112239980	112285930	−	2.04	6.4207E-04
*RP5-860F19.3*	Early B-cell factor 4	20	2612040	2688754	+	2.02	8.3191E-03
*RTN4RL1*	Reticulon 4 receptor-like 1	17	1786540	1787865	−	2.28	6.4207E-04
*SCUBE2*	Signal peptide, CUB domain, EGF-like 2	11	8998511	9069731	−	4.13	3.3991E-05
*SLITRK6*	SLIT and NTRK-like family, member 6	13	85264923	85271484	−	3.57	6.4207E-04
*STARD10*	StAR-related lipid transfer (START) domain containing 10	11	72143422	72182398	−	3.16	3.2803E-04
*TFF1*	Trefoil factor 1	21	42655462	42659713	−	3.38	1.3021E-03
*TMEM16A*	Transmembrane protein 16A	11	69602056	69713281	+	2.24	6.1384E-03
*TNFRSF21*	Tumor necrosis factor receptor superfamily, member 21	6	47307227	47385321	−	−2.07	1.6305E-03
*TSPAN13*	Tetraspanin 13	7	16785818	16789424	+	2.34	1.1854E-02
*UBD*	Ubiquitin D	6	29667268	29671562	−	−2.27	2.9004E-02

**Table 2:** Over-represented GO terms in the direct or one-hop interactors of proteins encoded by the gene-sets #1 and #2

	**Gene-set #1**	**Gene-set #2**
	**(*n*=198)^a^**	**(*n*=632)^a^**
**GO term**	**Number of interactors (FDR *P*-value)^b^**	**Number of interactors (FDR *P*-value)^b^**
Cell cycle	159 (<10^−9^)	267 (<0.001)
Cell death	138 (<10^−9^)	261 (<0.001)
Immune response	Nonsignificant	191 (<0.001)
Steroid hormone receptor signaling pathway	28 (<10^−10^)	Nonsignificant

a Number of gene products in the interactome network.

b Total number of interactors and corresponding FDR-adjusted *P-*value for GO term over-representation.
