# Supplementary figures and images for: Gene expression profiling integrated into network modelling reveals heterogeneity in the mechanisms of BRCA1 tumorigenesis
Source: Br J Cancer. 2009 Oct 13;101(8):1469–80. doi: 10.1038/sj.bjc.6605275 (PMC2768459; doi:10.1038/sj.bjc.6605275)

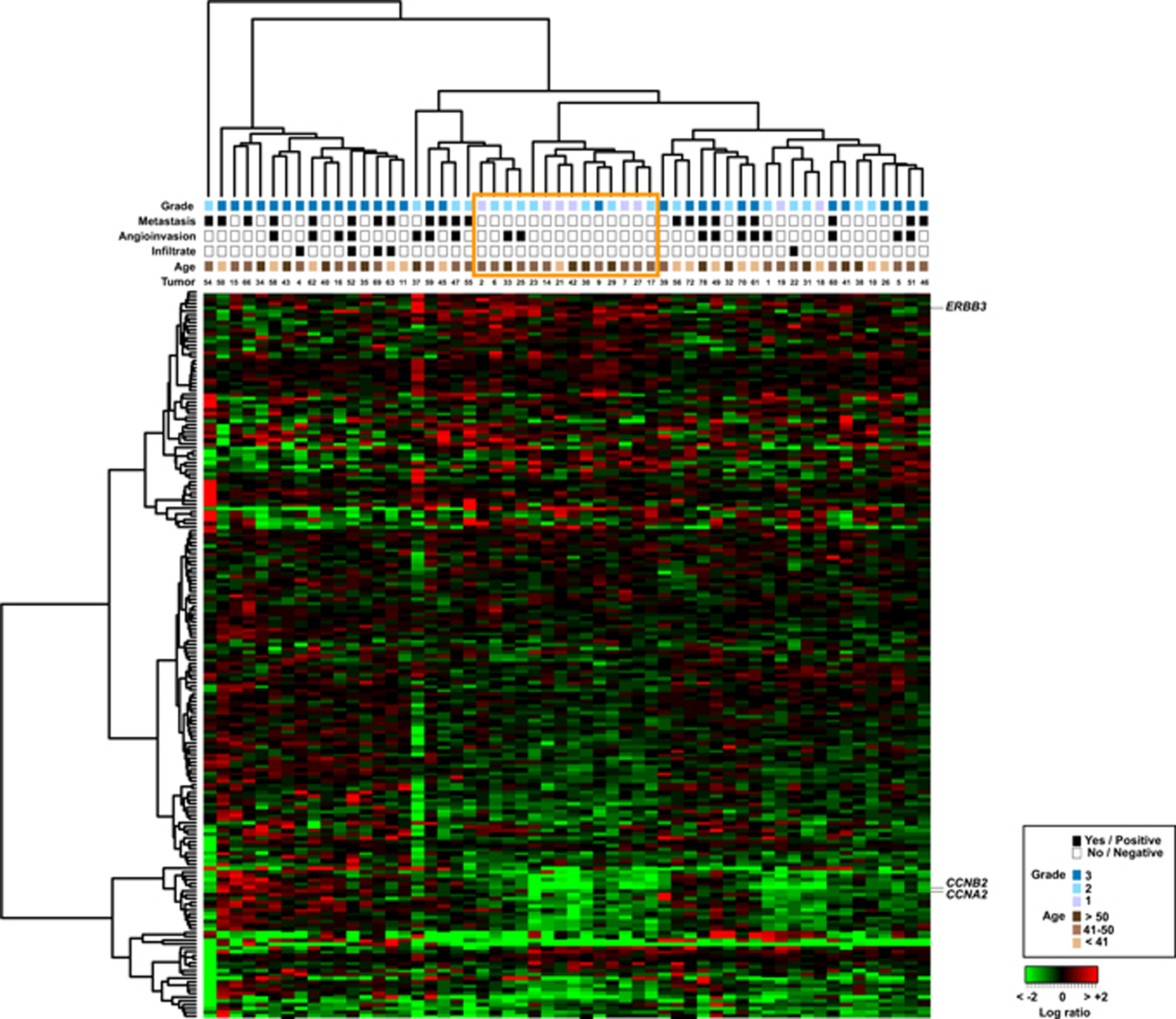

Supplement: Supplementary Figure S1 [file 6605275x1.tif]

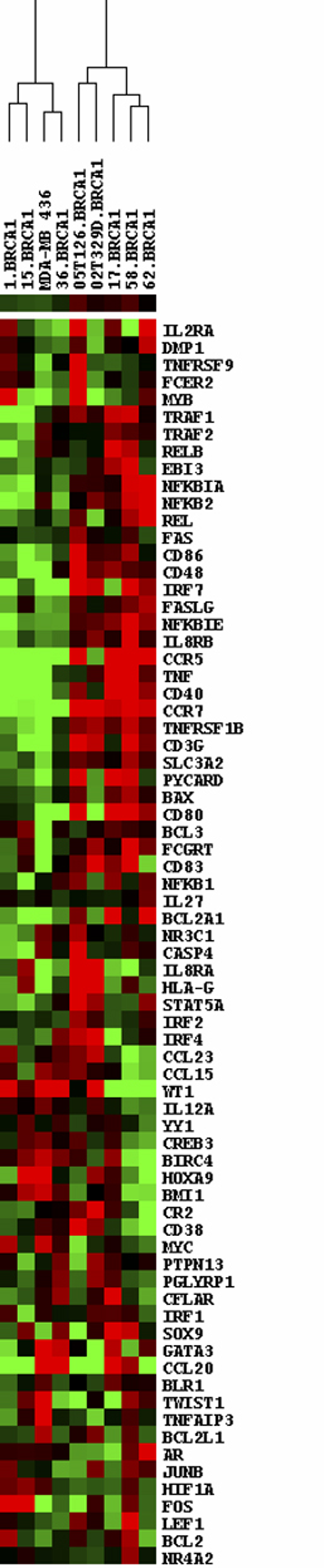

Supplement: Supplementary Figure S2 [file 6605275x2.tif]
