# Supplementary material for: Gene expression profiling integrated into network modelling reveals heterogeneity in the mechanisms of BRCA1 tumorigenesis
Source: Br J Cancer. 2009 Oct 13;101(8):1469–80. doi: 10.1038/sj.bjc.6605275 (PMC2768459; doi:10.1038/sj.bjc.6605275)
Supplement: Supplementary Figure Legends [file 6605275x3.doc]

**Supplementary figures legends**

**Supplementary figure S1.** Unsupervised hierarchical clustering obtained with the gene-set #1 in 56 ER-positive tumors from the external series of van’t Veer and colleagues (van’t Veer et al, 2002). . Within the orange square are included tumors with good prognosis showing down-regulation of cell cycle-related genes (*CCNA2 and CCNB2*) and over-expression of *ERBB3*. Clinical and pathological characteristics are annotated as shown in the inset and expression values are represented as log2 ratios.

**Supplementary figure S2.** Unsupervised hierarchical clustering of the ESR1-negative tumors and the BRCA1 mutant cell line MDA-MB 436 using 72 NFκB target genes related to apoptosis and immune system. Subgroups A and B are observed.
