# Supplementary material for: Gene expression profiling integrated into network modelling reveals heterogeneity in the mechanisms of BRCA1 tumorigenesis
Source: Br J Cancer. 2009 Oct 13;101(8):1469–80. doi: 10.1038/sj.bjc.6605275 (PMC2768459; doi:10.1038/sj.bjc.6605275)
Supplement: Supplementary Table S4 [file 6605275x7.doc]

**Supplementary table S4.** Histopathological and clinical data of the 14 BRCA1 tumors included in the analysis.

| **Tumor** | **Tumor type** | **Grade** | **ER** | **PGR** | **HER2**^§^ | **Other tumors in the probandus** | **Germline mutation**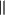, |
| --- | --- | --- | --- | --- | --- | --- | --- |
| X1 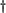 | IDC | III | Pos* | Pos* | Neg | Bil BC + OC 53 years^¶^ | 3522C>T (Q1135X)^f^ |
| X3 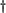 | IDC | III | Neg* | Neg* | Neg | No | 1499insA (479ter)^**^ |
| X15 | IDC | III | Neg* | Neg* | Neg | No | 4023G>T (E1302X)^**^ |
| X17 | IDC | III | Neg* | Pos* | Neg | No | P727L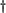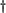,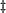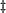 |
| X28 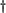 | IDC | III | Neg* | Neg* | Neg | Pancreatic Cancer at 67 years | 1499insA (479ter)^**^ |
| X33 | IDC | III | Pos* | Pos* | Neg | Bil BC | 4035delTT (1328ter)^**^ |
| X36 | IDC | III | Neg* | Neg* | Neg | No | 1100delAT (328ter)^**^ |
| X58 | IDC | III | Neg* | Neg* | Neg | Bil BC | 1100delAT (328ter)^**^ |
| X62 | IDC | III | Neg* | Neg* | Neg | Bil BC | 300T>G (C61G)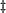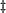 |
| X04T91 | NA | NA | NA | NA | NA | No | 3887delAG^**^ |
| X05T126 | IDC | III | Neg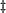 | Neg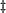 | NA | No | A1708E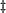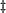 |
| X02T144 | NA | NA | NA | NA | NA | No | 3598del11 (1163 ter)^**^ |
| X02T329D | IDC | III | Neg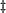 | Neg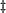 | Neg | No | IVS6-1C/T |
| X02T329I | IDC | III | NA | NA | Pos | No | IVS6-1C/T |

IDC: Infiltrating Ductal Carcinoma, NA: Information Not Available

* Receptor measured through Quantitative RT-PCR and immunohistochemical assay (a case was considered positive for ER and PGR when > 10% of the cells were stained)


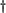
 #28 and #3 were classified as non-basal by expression profiling but as triple negative by immunohistochemistry. This discrepancy could be due to the presence of mRNA of *ESR1*, *PRG* and *ERBB2* in these tumors but lack of expression at the protein level. Interestingly, in the case of#28, the level of mRNA measured by RT-Quantitative PCR for *ESR1* was the highest and close to those of the tumors classified as positive by IHQ (data not shown). #1 was classified as *ESR1* negative but was positive when measured by Quantitative RT-PCR and immunohistochemical assay.


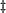
Receptor measured through immunohistochemical assay only.

^§^ According to the four category 0, +, ++, +++ DAKO system proposed for the evaluation of the HercepTest, only cases categorized in the +++ group were considered as positive.

^¶^Bil BC: Bilateral Breast Cancer, OC: Ovarian Cancer.


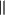
 Nomenclature of the mutations as described in the Breast Cancer Information Core: <http://research.nhgri.nih.gov/bic/>.

^**^ Mutations that by their localization in the central portion of the *BRCA1* gene are thought to activate the nonsense-mediated mRNA decay mechanism (NMD). For the 3887del11 mutation activation of NMD has been experimentally demonstrated (57).


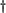

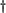
The P727L variant is considered a variant of unknown clinical significance.


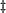

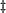
 Mutations that do not cause premature stop codons and do not activate NMD.
